# Mitochondrial plasticity supports proliferative outgrowth and invasion of ovarian cancer spheroids during adhesion

**DOI:** 10.3389/fonc.2022.1043670

**Published:** 2023-01-16

**Authors:** Joseph P. Grieco, Stephanie L. E. Compton, Nazia Bano, Lucy Brookover, Anna S. Nichenko, Joshua C. Drake, Eva M. Schmelz

**Affiliations:** ^1^ Graduate Program in Translational Biology, Medicine, and Health, Virginia Tech, Blacksburg, VA, United States; ^2^ Department of Human Nutrition, Foods and Exercise, Virginia Tech, Blacksburg, VA, United States

**Keywords:** mitochondria, mitophagy, mitobiogenesis, ovarian cancer metabolism, reoxygenation, spheroid, cycloheximide, adhesion

## Abstract

**Background:**

Ovarian cancer cells aggregate during or after exfoliation from the primary tumor to form threedimensional spheroids. Spheroid formation provides a survival advantage during peritoneal dissemination in nutrient and oxygen-depleted conditions which is accompanied by a suppressed metabolic phenotype and fragmented mitochondria. Upon arrival to their metastatic sites, spheroids adhere to peritoneal organs and transition to a more epithelial phenotype to support outgrowth and invasion. In this study, we investigated the plasticity of mitochondrial morphology, dynamics, and function upon adhesion.

**Methods:**

Using our slow-developing (MOSE-L) and fast-developing (MOSE-LTICv) ovarian cancer models, we mimicked adhesion and reoxygenation conditions by plating the spheroids onto tissue culture dishes and changing culture conditions from hypoxia and low glucose to normoxia with high glucose levels after adhesion. We used Western Blot, microscopy and Seahorse analyses to determine the plasticity of mitochondrial morphology and functions upon adhesion, and the impact on proliferation and invasion capacities.

**Results:**

Independent of culture conditions, all spheroids adhered to and began to grow onto the culture plates. While the bulk of the spheroid was unresponsive, the mitochondrial morphology in the outgrowing cells was indistinguishable from cells growing in monolayers, indicating that mitochondrial fragmentation in spheroids was indeed reversible. This was accompanied by an increase in regulators of mitobiogenesis, PGC1a, mitochondrial mass, and respiration. Reoxygenation increased migration and invasion in both cell types but only the MOSE-L responded with increased proliferation to reoxygenation. The highly aggressive phenotype of the MOSE-LTICv was characterized by a relative independence of oxygen and the preservation of higher levels of proliferation, migration and invasion even in limiting culture conditions but a higher reliance on mitophagy. Further, the outgrowth in these aggressive cells relies mostly on proliferation while the MOSE-L cells both utilize proliferation and migration to achieve outgrowth. Suppression of proliferation with cycloheximide impeded aggregation, reduced outgrowth and invasion via repression of MMP2 expression and the flattening of the spheroids.

**Discussion:**

Our studies indicate that the fragmentation of the mitochondria is reversible upon adhesion. The identification of regulatory signaling molecules and pathways of these key phenotypic alterations that occur during primary adhesion and invasion is critical for the identification of druggable targets for therapeutic intervention to prevent aggressive metastatic disease.

## Introduction

According to the American Cancer Society, ovarian cancer currently ranks as the 5^th^ leading cause of cancer-related deaths in women with an overall survival rate of 50%. A recent estimation predicts 19,880 new diagnoses whereas 12,810 women will die from ovarian cancer in 2022 (1). The late detection of the disease contributes to a survival rate of less than 30% when the disease has spread (2). Ovarian cancer metastases can displace from primary tumors -either the ovaries or the fallopian tubes- during different stages of development, making ovarian metastasis a highly heterogeneous and genetically variable disease (3, 4). Almost all exfoliated ovarian cancer metastases follow a conserved pattern of transcoelomic dissemination, reaching the omentum within hours (3, 5). The peritoneal cavity’s microenvironment is a hostile environment, restricting cell viability and proliferation due to physical stresses (6), low oxygen concentrations (1-2%), and low glucose and glutamine levels (4). However, ovarian cancer cells are able to adapt to these conditions through aggregation which provides a survival signal during primary metastasis (7, 8). Upon adherence to the omentum, spheroids undergo mesenchymal-epithelial transition to aid outgrowth and basal membrane invasion to regain access to nutrients and oxygen (9). The molecular mechanisms involved in supporting survival in disseminating spheroids but also in promoting adhesion, invasion and outgrowth at secondary sites remain unclear. Previous studies in our lab showed that ovarian cancer cells become more glycolytic during progression and aggregation (10–12). Aggregation of the cancer cells was accompanied by a fragmented mitochondrial phenotype especially in the core of our spheroid models (13) resulting in a drastic reduction of respiration (10) as well as increased mitophagic signaling but not an induction of apoptosis (Grieco et al., under review). We have further shown a change in cellular metabolism in the earliest stages of spheroid adhesion (14), suggesting that successful ovarian metastases can adapt to the changing conditions they are exposed to from exfoliation, dissemination, secondary site attachment to invasion and induction of angiogenesis without loss of viability.

Mitochondrial dynamics are consistently regulated to support bioenergetic and biosynthetic processes and, thus, are constantly adapting to changes in the environment (15). Mitochondria are not only key responders of energy and metabolic functioning, but also play a key role in signal transduction, cell proliferation and apoptosis (15, 16). The organelles themselves are internally regulated *via* mitochondrial quality control mechanisms including mitochondrial dynamics, mitophagy and mitobiogenesis (17). Mitochondrial dynamics (process of fission and fusion) regulate mitochondrial morphology and their networking capacity but are also important in facilitating mitochondrial content sharing between individual mitochondria (16). Imbalanced mitochondrial fission supports a more fragmented and depolarized phenotype in benign cells that can result in mitophagy induced cell death (18) which is not activated in cancer cells as previously seen in our murine ovarian cancer cell model (13). Mitophagy, the selective removal of depolarized mitochondria, can be induced as a result of increased damaged mitochondria from increased respiratory activity and reactive oxygen species (ROS) production (19, 20). Hypoxia has been shown to induce mitophagy in breast, lung, and cervical tumors (21), ovarian cells (13) and in a variety of tumor cells during dissemination (see recent review (22)); this is thought to be a mechanism to support viability. However, oxygen supplementation after hypoxia (reoxygenation) activated mitophagy in astrocytes (23), kidney cells (24) and cardiac muscle (25) and is thought to protect the tissues against reperfusion damage (26). This may be cell-type specific since a reduction in mitophagy was observed in reoxygenated intestinal cells; concurrent with the observations in other tissues, the lack of mitophagy contributed to cellular damage due to the disturbance of mitochondrial homeostasis and subsequent induction of apoptosis (27) indicating that mitophagy is critical to prevent cell and tissue injury after reoxygenation. The mechanisms behind the adaptation to changing oxygen levels have not been clearly elucidated in cancer cells but in contrast to cardiomyocytes, an increase of mitophagy was not observed in HeLa cells after 6 h of reoxygenation (28).

Mitobiogenesis has been shown to promote proliferation and tumor growth *via* an increase in energetic production (29–31). Peroxisome proliferator-activated receptor gamma coactivator 1 alpha (PGC1α)-mediated mitobiogenesis in breast cancer cells supports migratory and invasive capacity *via* enhanced oxidative phosphorylation (32). This process initiates with PGC1α coactivating nuclear respiratory factors 1 and 2 (NRF1/2) to promote expression of mitochondrial transcription factor A (TFAM) aiding in the synthesis of mitochondrial DNA transcripts and translation of key oxidative/glycolytic enzymes (see recent review (33). The link between mitobiogenesis and reoxygenation has yet to be elucidated.

In the present study, we investigated the plasticity of mitochondrial morphology, dynamics and functions after adhesion. We evaluated early adhesion events -the stimulation of proliferation, migration, and invasion - in ovarian spheroids over time and in response to re-oxygenation as would be expected after the cancer cells are reaching existing blood vessels or inducing angiogenesis. In addition, we assessed how this would contribute to a shift in the balance of mitobiogenic and mitophagic signaling. Using the extensively characterized mouse ovarian surface epithelial (MOSE) cell model for progressive ovarian cancer (7, 34–37), we show differences in the functional adaptations in slow- (MOSE-L) and fast-developing MOSE-L_TIC_ *_v_* serous ovarian cancer spheroids that may be contributing to the aggressive tumor-initiating phenotype of the MOSE-L_TIC_ *_v_*. Our results show that the fragmented mitochondrial phenotype is reversible upon adhesion, suggesting that the plasticity of the mitochondrial phenotype is critical for the adaptation to the changing conditions during ovarian metastasis. Identifying the molecular mechanisms of this mitochondrial plasticity, the reversal of fragmentation as the mitochondria adapt to environmental conditions, is critical for the development of novel treatments that could suppress ovarian cancer metastasis.

## Methods

### Cell culture

The mouse ovarian surface epithelial (MOSE) cell model of progressive serous ovarian cancer has been previously characterized (7, 34–37). Briefly, For the present studies we used cells that differ in their capacity to induce lethal disease. MOSE-L_TIC_ *_v_* represent fast-developing disease which only need 1x10^3^ cells to reach the endpoint while MOSE-L cells need 1x10^6^ cells and ~100 days to achieve the same and therefore represent slow-developing disease (7, 34, 36). Cells were routinely grown in optimal growth media (high glucose Dulbecco Modified Eagle Medium, 27mM) (HG DMEM, Sigma), 4% fetal bovine serum (FBS), 3.7g/L sodium bicarbonate, 110mg/L sodium pyruvate and 1% penicillin-streptomycin at 37°C with 21% O_2_ and 5% CO_2_.

To generate spheroids of a uniform size, cells were seeded in ultra-low adherent 96-well plates (Corning) at a concentration of 5x10^3^, 2x10^4^ or 1x10^6^ cells/well as described (10, 13). The cells were incubated in limiting media conditions (low glucose DMEM; 5 mM) (LG DMEM, Sigma), 1% FBS, and 1-2% O_2_ for 24 h. Some spheroids were transferred to a tissue culture-treated 96-well dish and allowed to adhere for 0, 4, 8 and 12 h in limiting media conditions. After 12 h, adherend spheroids were either exposed to normoxia (reoxygenated - reox) and optimal growth medium or were left in limiting conditions (non-reox, LG) for the indicated time periods; a medium change was also performed for the latter to eliminate differences due to fresh medium.

### Outgrowth imaging

MOSE-L and MOSE-L_TIC_ *_v_* spheroids (5x10^3^ cells/spheroid) were allowed to adhere for the indicated time points, culture conditions and treatments. Images were taken at 10x magnification for each timepoint on a standard light microscope (Nikon) to assess the outgrowth from edges of the spheroids. Outgrowth measurements were quantified using ImageJ, by averaging 3 individual measurements of diameter of the outgrowth from the base of the spheroid to the end of the outgrowth layer for each spheroid (mean diameter of outgrowth).

### Alamar blue assay

Spheroids (5x10^3^ cells/spheroid) were placed into a 96-well assay plate (Corning) in both reox and non-reox conditions and adhered for the indicated time points. Adherent spheroids were incubated with alamar blue cell viability reagent (Invitrogen) 4 h before the assay plate was read with a standard plate reader (BioTek). Plates were read at 600 nm and 570 nm excitation and emission, respectively to quantitate the oxidation: reduction ratio indicative of the spheroid’s proliferative capacity.

### Invasion assay

Spheroids were embedded in collagen gels consisting of 2 mg/mL collagen type I (Corning), 10x DMEM, 1N NAOH and deionized water and incubated in 37°C for 1 h. Subsequently, spheroids were placed in limiting and optimal growth conditions for 9 d and 14 d with media changes biweekly. Spheroids were qualitatively analyzed for ability to invade collagen layer using standard light microscopy at 10x magnification (Nikon). This method was repeated for drug-treated spheroids reoxygenated for 24 and 36 h.

### Migration assay (Boyden chamber)

Single spheroids were placed on 8 µm pore inserts (Falcon) in serum-free medium for 4 h in 24-well companion plates (Falcon) and treated as indicated. Once adherent, HG medium with 4% FBS as chemoattractant was added in the bottom of each well and spheroids were either placed in normoxic (21% O_2_) or hypoxic (1-2% O_2_) conditions for 6 h. Spheroids were then gently removed washed from the top layer of the insert with PBS. Each insert was fixed in 100% ice-cold methanol for 15 min at -20 °C, washed 2x with PBS for 5 min and then mounted onto glass slides with Gold Antifade Mounting media with DAPI (ThermoFisher) and covered with 2 mm glass coverslips. Cells were imaged and counted using a Nikon 80*i* fluorescent microscope for each spheroid that was placed in the insert.

### Western blotting

Adherent spheroids harvested at the indicated timepoints and culture conditions were lysed in radioimmunoprecipitation buffer supplemented with protease and phosphatase inhibitor tablets (ThermoFisher). Protein concentrations were determined using the Pierce Bicinchoninic Acid assay (ThermoFisher), normalized to 1 µg/µL and mixed with 50:1 2x Laemmli sample buffer (BioRad): 2-mercaptoethanol (ThermoFisher). Samples were run in a 10% acrylamide SDS gel and transferred onto a PVDF membrane (BioRad). Total protein was used to normalize individual proteins as was quantified using the total protein normalization substrate (ThermoFisher). Blots were blocked with 5% milk in 1X Tris-buffered saline with 0.1% Tween 20. Primary antibodies against Tomm20 (Millipore), TFAM (Abcam), PGC1α (Millipore), BNIP3 (Abcam), LOX (Abcam), MMP2 (Novus Bio), and MMP9 (Novus Bio), and LC3B (Cell Signaling) were imaged using IRDye 680 cw rabbit and 800 cw mouse secondary antibodies (LiCor) and the Licor Odyssey CLx Imager. Proteins were quantified and normalized to total protein using ImageJ.

### Immunofluorescence staining

MOSE cells were incubated with 50 nM MitoTracker Red CMXRos (Molecular Probes) and placed in ultra-low adherent dishes to form single spheroids. Formed spheroids were placed into 35 mm glass bottom dishes (Cellvis) for 16, 20 and 24 h followed by fixation for 15min with cold methanol. Fixed spheroids and outgrowth (cells that were growing mostly as monolayers on the culture dish) were subsequently probed with LC3B (Cell Signaling) and PGC1α (Millipore) and costained with DAPI for identification protein-nuclear localization. Outgrowth cells were imaged at 40 x magnification using an Opterra inverted confocal microscope. Proteins were analyzed for Corrected total cell fluorescence intensity using ImageJ. Images were processed using Adobe Photoshop CS6.

### Mitochondrial respiration (Seahorse XFe96 analyzer)

Mitochondrial respiration in spheroids was measured using the Seahorse XFe96 extracellular flux analyzer (Agilent). Spheroids were seeded at 2x10^4^ cells/well and transferred to XFe96 cell culture plates for the specified adherent timepoints and drug treatments. Prior to experimental procedures, the cellular media was replaced to serum- and bicarbonate-free medium. Oxygen consumption rate (OCR) measurements including basal, maximum respiration, spare-respiratory capacity, ATP synthesis, proton leak, and non-mitochondrial respiration were obtained and calculated as previously described (10, 14). Spheroid respiration measurement cycles consisted of 3 min mixing, 2 min wait, and 3min measurement cycles. Inhibitor concentrations used for spheroids were predetermined in preliminary studies to include 1.0 µM/L oligomycin, 2.0 µM/L carbonyl-cyanide-p-trifluoromethoxy-phenylhydrazone, and 1.0 µM/L rotenone/antimycin A (10).

### MitoTimer transfection/analysis

To assess mitochondrial oxidative modifications in spheroids that are indicative of the quality of mitochondria and sometimes interpreted as mitochondrial age in cancer cells, we used MitoTimer-transfected cells as previously described (38). MitoTimer plasmids (Addgene) were grown in *E. Coli* vectors using ampicillin-infused nutrient broth media and agar plates (Fisher BD Difco™). Bacterial colonies were incubated at 37°C overnight. Plasmids were purified using the PureLink™ HiPure Plasmid Maxiprep kit (ThermoFisher). Transfection of MitoTimer plasmids was conducted on adherent MOSE-L and MOSE-L_TIC_ *_v_* cells seeded in a 6-well format at 5x10^5^ grown in serum-free Opti-MEM medium (ThermoFisher). Lipofectamine 2000 DNA Transfection Reagent (Invitrogen) and plasmid DNA were separately diluted in serum-free Opti-MEM media and combined at a 1:3 ratio (4 µg DNA:12 µL Lipofectamine) incubating at room temperature for 30 min. The DNA-lipid complex was then added to each well and placed in 37°C, 5% CO_2_ incubators for 4 h followed by 10% FBS addition for 20 h. Identification of transfection efficacy was performed with a Nikon fluorescent inverted scope with TRITC and FITC filters. Cells were then seeded at 5x10^3^ cells/well in ultra-low adherent conditions overnight and then placed on 35 mm glass-bottom dishes (Cellvis) for 16-24 h in the indicated conditions. Spheroids were imaged using FITC and TRITC Lasers and reconstructed using 20 Z-stack slices of 5µm length on a Leica DMI8 MP confocal and Leica LASX analysis software. The direct site of adherence on each spheroid was analyzed for fluorescence intensity using the Leica LASX analysis software. MitoTimer quantifications were conducted using previously established Matlab-based algorithm as previously described (39, 40).

### MTT (3-[4,5-dimethylthiazol-2-yl]-2,5 diphenyl tetrazolium bromide) assay

Cells were seeded at 2.5x10^3^ cells/well and incubated overnight. Cycloheximide (Cyclo, protein translation/proliferation inhibitor, Sigma) and Latrunculin A (Lat A, actin stabilizer, Calbiochem) were added at 0, 50, 100, 200, and 500 nM concentrations for Cyclo and 0, 1, 5, 10, and 20 nm concentrations for Lat A for an additional 24 h incubation. Thiazolyl Blue Tetrazolium Bromide MTT solution was then added for 3 h followed by lysis of the cells. Absorbance was read at 570nm. For Cyclo, a value of 50% compared to control was considered satisfactory for blocking most of the proliferative capacity. For Lat A, a % viability greater than 80% was deemed necessary to avoid the impact of cell death on the measurements.

### Spheroid formation assay

MOSE cells were seeded at 5x10^3^ in ultra-low adherence plates in medium supplemented with 100 nM Cyclo, 10 nM Lat A, or both for 24 h. Aggregation capacity was determined qualitatively by imaging at 10 x magnification on a standard light microscope (Nikon).

### Bafilomycin A1 treatment

Cells were seeded in flat-bottom 96-well dishes, incubated for 24 h and then treated with 100 nM Bafilomycin A1 (BafA1) for 24h. Viability was measured by alamarBlue assay (13). To determine the impact of BafA1on spheroids, cells were seeded in ultra-low adherence round bottom 96well dishes and treated either immediately (0h) with 100 nM BafA1 for 24 h, or after 24 h when spheroids had already formed for an additional 24 h. Viability was determined by alamarBlue assay as described (27). Images were taken before adding the alamarBlue.

### Cell tracker assay

Spheroids were live-stained with 5 µM cell-tracker 5-Chloromethylfluorescein diacetate (CMFDA) (ThermoFisher) for 45 min at 37°C followed by fresh media replacement with Cyclo, Lat A, or no inhibitors. This procedure allows for the permeation of the CMFDA through the spheroid (13). After staining, single spheroids were placed onto 35mm Cellvis dishes for 24 h. Spheroids and outgrowth were imaged using a Leica DMI8 MP confocal microscope at 25 X magnification. Additional tile scans were used to capture the entirety of the outgrowth from the spheroid of MOSE-L_TIC_ *_v_* control and Lat A groups using the LASX software.

### Spheroid spreading imaging and quantification

To assess the conformational changes of the spheroids, we incubated EGFP-transfected spheroids adherent for 4 h with the indicated drugs for 24 h and generated 3D reconstructions from 100µm stacks (see above) on a Leica DMI8 MP confocal microscope. Top- and side-view stack views were assessed and depth coded to identify which layers of the spheroid were occupying different regions of the field of view, indicating spread and representing changes in conformation of the primary spheroid. Quantification of height and width of spheroids was conducted using ImageJ.

### Immunofluorescence staining of outgrowth

Spheroids were adhered onto collagen gels consisting of 2 mg/mL collagen type I (Corning), 10x DMEM, 1N NAOH and deionized water as described above (see invasion assay). Spheroids adhered for 14 d and were subsequently fixed with 100% methanol. Invaded outgrowth cells were stained with MMP2 and LOX primary antibodies and FITC and TRITC-conjugated secondary antibodies respectively. MMP2 and LOX expression were imaged using a Leica DMI8 MP confocal microscope.

### Statistics

Data are presented as mean ± SEM with at least 3 biological replicates. Cell types were compared independently or compared by one-way ANOVA. Individual unpaired t-tests for a specific timepoint or one-way ANOVA were used to identify changes over time.

## Results

Even short-term adhesion alters cellular metabolism (14), suggesting that the mitochondrial phenotype observed in the spheroids -fragmented with very low respiration- is reversible upon adhesion. Here we mimicked early secondary metastatic outgrowth conditions and investigated if differential mitochondrial plasticity is supporting the adaptation to changing culture conditions that may contribute to the differing metastatic capacity of MOSE-L and MOSE-L_TIC_ *_v_* cells.

### Reoxygenation supports growth and outgrowth in slow-developing adherent spheroids

**Figure 1:**
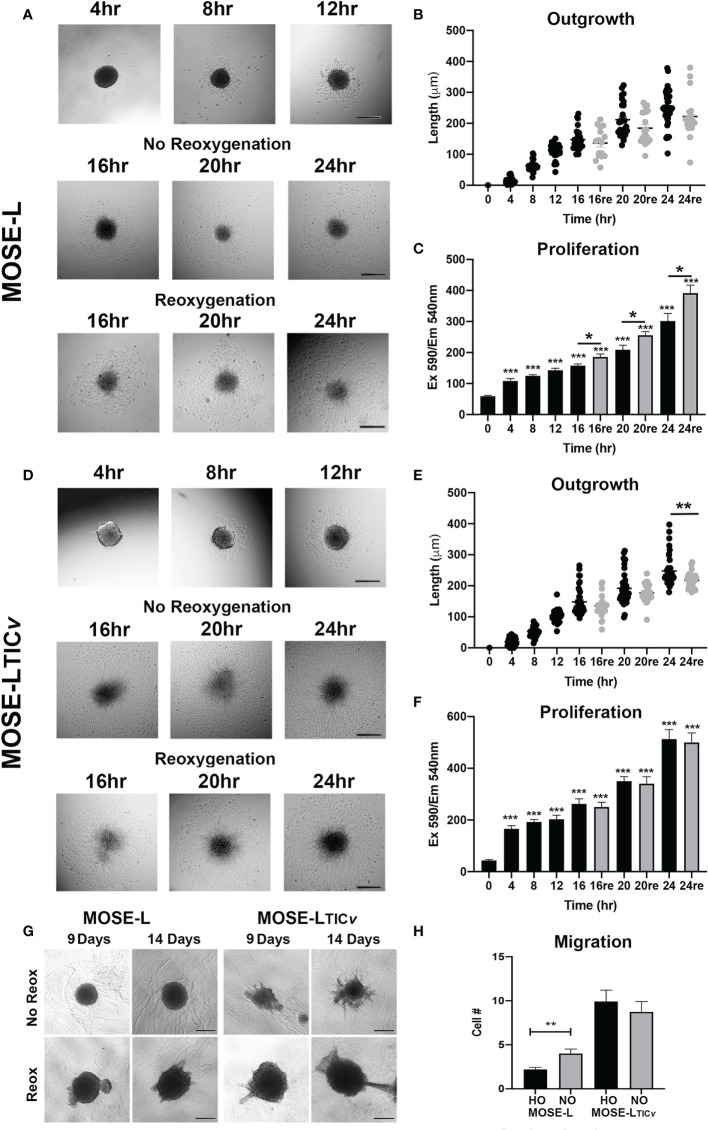
Adhesion and reoxygenation (reox) promote proliferation, invasion and migration. MOSE-L **(A–C)** and MOSE-L_TIC_ *_v_* **(D–F)** spheroids adhered to culture plates in non-reoxygenated conditions and were switched to reoxygenated (re) conditions after 12 h. **(G)** Spheroid invasion into collagen gels after 9 and 14 days; **(H)** Boyden chamber analysis of cellular migration in normoxic (NO) and hypoxic (HO) conditions. Different from control at *p<0.05, **p<0.01, ***p<0.001. Scale bar set at 200µm.

Mimicking adhesion of the disseminating cancer spheroids to secondary sites, MOSE spheroids were plated onto tissue culture dishes. During a time-course of 0-24 h, both MOSE-L and MOSE-L_TIC_ *_v_* adhered to and grew out onto the tissue culture dishes; after an initial adhesion period of 4 h with very little outgrowth, there was a significant increase in outgrowth between each 4 h interval (4-24 h, p<0.01 for both cell lines) (**Figures 1A, B, D**). Accompanying the outgrowth was a significant initial increase in proliferation at 4 h of adhesion in both cell types (p<0.05) which did not significantly further increase until 16 h of adhesion (**Figures 1A–D**). To determine if access to oxygen and nutrients would support outgrowth, we changed the culture conditions from limiting conditions to serum-supplemented HG medium and normoxia after 12 h of adherence. This reoxygenation (reox) reduced the outgrowth capacity of both spheroid types which was significant for the MOSE-L_TIC_ *_v_* spheroids after 24 h of adhesion (p<0.01) (**Figures 1C**). MOSE-L_TIC_ *_v_* spheroids showed a significantly faster adhesion and outgrowth than MOSE-L at 4 and 8 h (p<0.05) under limiting conditions; at later time points or in reox conditions no significant differences between the two cell lines were observed, suggesting differences in their metastatic potential is not determined by adhesion and outgrowth capacity. Interestingly, we found that reox only significantly increased the proliferation of the MOSE-L spheroids (p<0.05) while there was no effect on the MOSE-L_TIC_ *_v_* spheroids at any timepoint (**Figures 1C**). Compared to the MOSE-L spheroids, MOSE-L_TIC_ *_v_* spheroids were growing significantly faster at all timepoints and in both culture conditions than the MOSE-L spheroids (P<0.05 for 24 h reox, p<0.01 for 16 and 20 h reox and p<0.001 for all other).

We next investigated how reox conditions affect invasion of embedded spheroids in collagen matrices. As shown in **Figure 1**, the MOSE-L spheroids increased their size in both conditions but only were capable of invading the collagen gels in reox conditions while the MOSE-L_TIC_ *_v_* spheroids exhibited a proliferative and invasive phenotype regardless of culture conditions. Finally, we assessed migratory potential in limiting vs. optimal conditions and found that only the MOSE-L spheroids were responsive to the culture conditions and significantly increased migration when both glucose and oxygen were present (p<0.01) (**Figure 1**) while MOSE-L_TIC_ *_v_* spheroids had a significantly higher migratory capacity than the MOSE-L spheroids regardless of oxygen content (p<0.01 for normoxia and p<0.001 for hypoxia). Together, these data indicate that both cell types are capable of adhesion, migration and invasion in hypoxic and nutrient-deprived conditions; these functions are enhanced in the MOSE-L spheroids after access to nutrients and oxygen while the more aggressive MOSE-L_TIC_ *_v_* spheroids are mostly independent of culture conditions. Our data also suggest that the overall rate of proliferation is not determined by the outgrowth size; instead, the proliferation increase may only be apparent at the adhesion site while the bulk of the spheroid may not respond to the adherence signal with increased proliferation at the chosen time points. This is in agreement with our observation that polarized mitochondria are highly visible at adhesion sites but not in the spheroid core (Grieco et al., 2022).

### Changes in mitobiogenesis and mitophagy protein expression are early events during adhesion and reoxygenation

**Figure 2:**
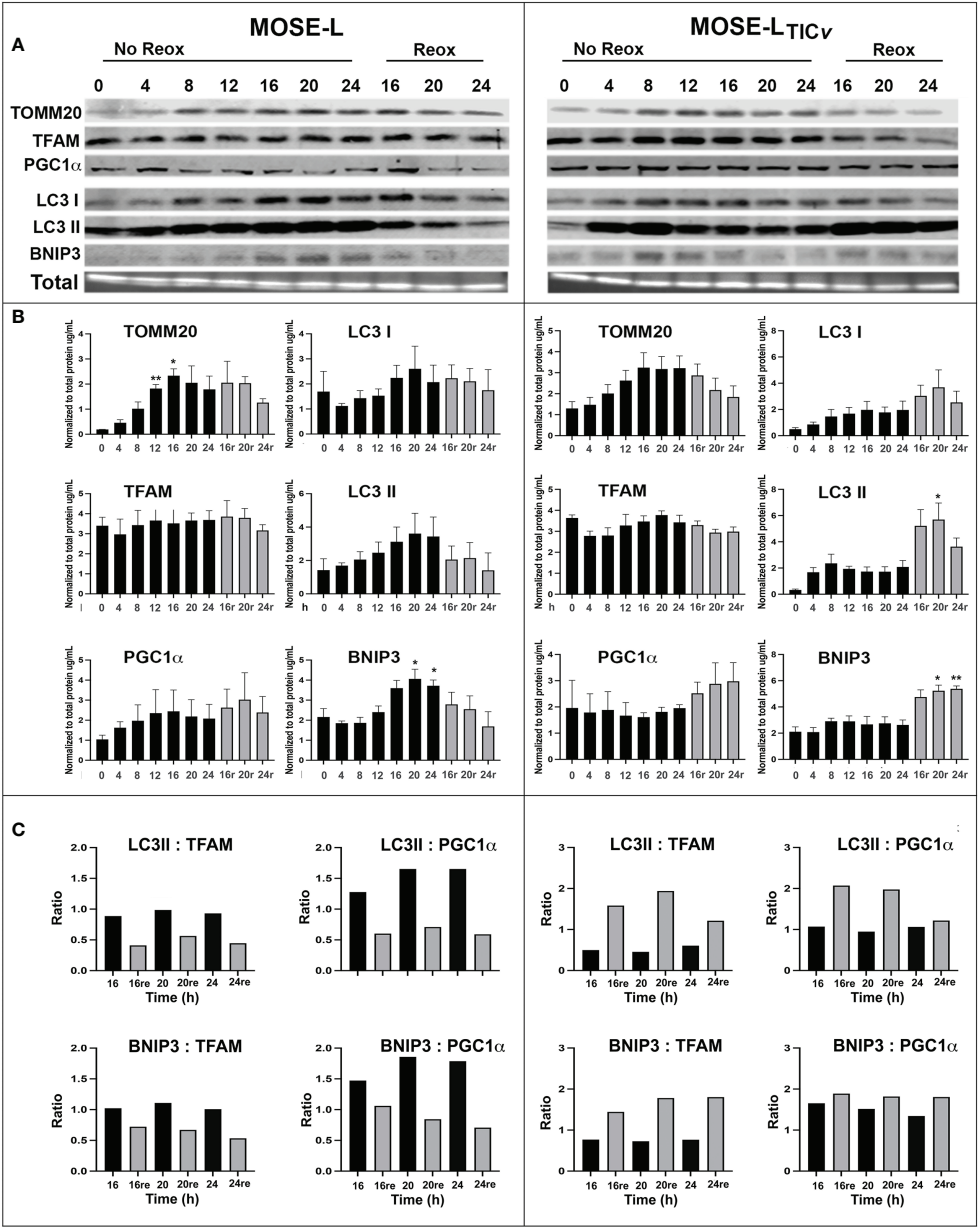
Mitochondrial quality control proteins are altered during reoxygenation. **(A, B)** Western blot for MOSE-L and MOSE-L_TIC_ *_v_* spheroids adherent for 0-24 h in non-reox vs. reox (16-24 h) conditions. **(C)** Ratios of mitophagy: mitobiogenesis proteins comparing reox vs. non-reox spheroids. *p<0.05 *vs.* control **p<0.05.

Hypoxia and aggregation induced mitophagy in the MOSE spheroids (13); however, upon adhesion, the upregulation of mitochondrial mass and energy production is needed to support accelerated proliferation, migration and invasion (29, 32). Therefore, we next investigated how proteins involved in hypoxia-mediated mitophagy and mitobiogenesis were regulated during the early stages of adhesion and in response to reoxygenation. We found that the mitochondrial membrane protein TOMM20 that has been used as mitochondrial mass indicator (41, 42) was significantly increased in MOSE-L cells after 12 and more hours of adherence. TFAM expression was stable while PGC1α expression increased in response to reox (**Figures 2A**). Additionally, the autophagy proteins BNIP3, LC3I and LC3II were increased by adhesion in non-reox conditions for the MOSE-L spheroids and decreased during reox. Similarly, mitochondrial content marker TOMM20 increased over time of adherence in MOSE-L_TIC_ *_v_* spheroids while there was little change in other proteins. However, we found that BNIP3, LC3I and LC3II were higher expressed in reox conditions in the more aggressive MOSE-L_TIC_ *_v_* spheroids where BNIP3 was significantly increased at 20 and 24 h compared to 0 h (p<0.05). The ratios between mitophagic and mitobiogenic proteins were calculated to determine if the culture conditions affect mitochondrial dynamics. The higher expression of mitophagic proteins in non-reox conditions and higher expression of mitobiogenic proteins in reox conditions ratio in MOSE-L spheroids suggest that these cells respond to oxygen and optimal medium with an increase in mitobiogenesis which correlates well with their higher proliferation in these conditions (**Figure 1**). In contrast, the MOSE-L_TIC_ *_v_* spheroids displayed a shift towards mitophagy in reox conditions apparent by the much higher expression levels of BNIP3 and LC3 (**Figure 2**). There was no association with their growth rate since MOSE-L_TIC_ *_v_* spheroids already grow faster than the MOSE-L spheroids without additional change after reox (see **Figure 1**). These data indicate a cell type-specific response to reoxygenation: while the MOSE-L cells that represent slow-developing disease still responded to growth conditions and began to reverse their mitophagic phenotype acquired during aggregation, the MOSE-L_TIC_ *_v_* were independent of culture conditions and increased mitophagy. This resilience to the cells’ environment has been shown to correlate with a more aggressive phenotype in disseminating breast, endometrial, and other cancer cells and their survival while suppressing cancer development in others (see recent review (43)). Our data suggest that this shift to mitophagy also may contribute to the more aggressive phenotype of the MOSE-L_TIC_ *_v_* spheroids.

**Figure 3:**
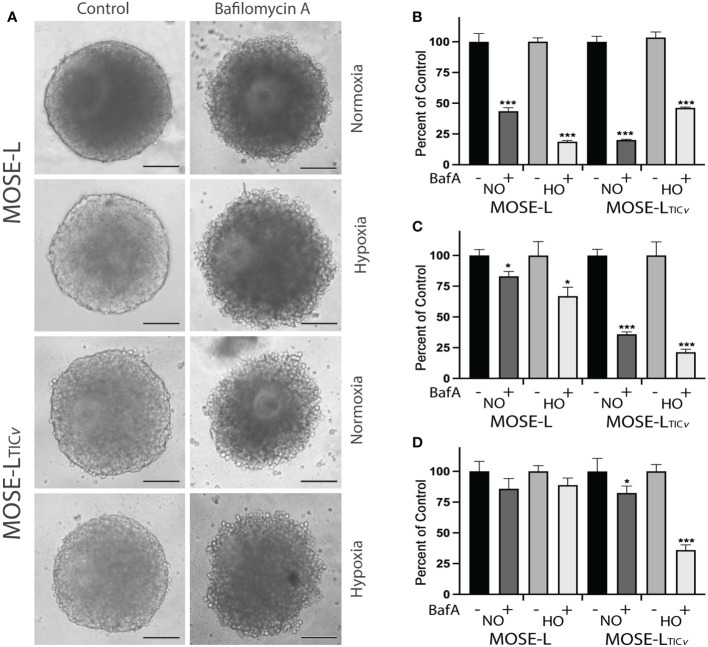
Inhibition of mitophagy affects cell viability. **(A)** Bafilomycin A1 (Baf1) treatment caused the deterioration of spheroids in normoxia (NO) and hypoxia (HO). **(B)** Adherent cells were treated with BafA1 for 24 h. **(C)** Reduced viability when cells were treated with BafA1for 24 h at time of seeding and **(D)** after the spheroids had formed. *p<0.05 and ***p<0.001 *vs.* untreated control.

To confirm the importance of mitophagy for the survival of the spheroids, we treated the spheroids with BafA1, a potent inhibitor of vacuolar-type H+ ATPase that inhibits autophagosome fusion with lysosomes and subsequently the acidification of autophagasomes and the degradation of mitochondria. BafA1 treatment significantly reduced the viability of adherent cells in both cell lines in normoxia and hypoxia (**Figure 3**). When the cells were treated with BafA1 at seeding, the spheroid formation was not affected (not shown). However, the spheroids began to deteriorate (**Figure 3**) and the viability was significantly reduced in both cell lines but significantly more in the MOSE-L_TIC_ *_v_* spheroids (P<0.001 in both normoxia and hypoxia compared to MOSE-L); hypoxia decreased viability further in the MOSE-L_TIC_ *_v_* (p<0.001) but not in the MOSE-L (**Figure 3**). When treated after spheroid formation, there was less of an effect on the MOSE-L spheroids but a reduced viability was observed in the MOSE-L_TIC_ *_v_* which was again more pronounced in hypoxia (p<0.001 *vs*. Baf1A-treated MOSE-L_TIC_ *_v_* in normoxia) (**Figure 3**). Thus, blocking mitophagy is especially deleterious to MOSE-L_TIC_ *_v_* cells, suggesting these cells rely on mitophagy for mitochondrial quality control and survival.

### Reoxygenation promotes functional and protein expression changes in the outgrowth of the spheroids

**Figure 4:**
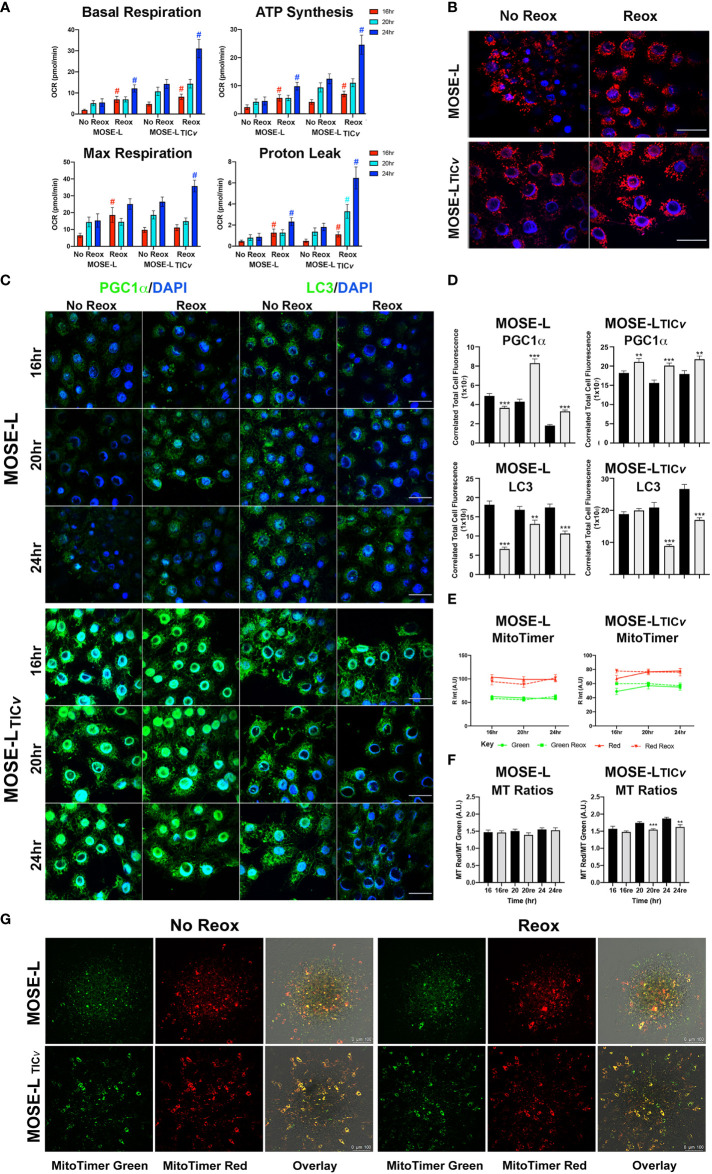
Adhesion-induced mitochondrial plasticity allows for adaptations to changed culture conditions. **(A)** Seahorse XFe96 analyses of cellular respiration; #p<0.05 reox group compared to non-reox counterpart for each timepoint and cell type. **(B)** Outgrowth imaging of cells stained with MitoTracker CMXRos and DAPI after 24 h adherence of spheroids in non-reox *vs*. reox conditions. **(C, D)** Outgrowth imaging and quantitation of 16-24 h spheroids immunostained with PGC1α/DAPI and LC3/DAPI. **(E)** MATLAB quantification of MitoTimer fluorescence intensity in reox *vs*. non reox conditions; green indicates mitobiogenesis, red indicates hydrogenated MitoTimer over time. **(F)** MitoTimer red: green fluorescence intensity ratios. **p<0.01 ***p<0.001vs. the non-reox counterparts. Scale bar set at 50µm. **(G)** MitoTimer expression in the outgrowth of adherent spheroids.

To determine if the increased proliferation after adhesion is supported by an increase in cellular ATP synthesis, we next determined how cellular respiration is affected by adhesion and reox conditions using a Seahorse XFe96 analyzer. Adhesion significantly increased the oxygen consumption rate (OCR) of both the MOSE-L and MOSE-L_TIC_ *_v_* spheroids over time, including basal respiration, ATP synthesis, and maximal respiration (**Figure 4**); basal respiration and ATP synthesis was higher in the MOSE-L_TIC_ *_v_* spheroids, correlating with their higher proliferation rate. The proton leak was also higher in the MOSE-L_TIC_ *_v_* spheroids, suggesting a mechanism to protect from ROS generated by oxidative phosphorylation. Further, switching to reox culture conditions significantly increased OCR in both cell types compared to non-reox groups. We also calculated spare respiratory capacity and glycolytic reserve and found that the spare respiratory capacity was significantly reduced in the reox MOSE-L_TIC_ *_v_* spheroids and glycolytic reserve was increased over time with no effect of the reox conditions. Non-mitochondrial respiration was significantly reduced only in reox MOSE-L compared to non-reox groups (p<0.05) (**Figure 1**). After 24 h, basal respiration (p<0.05), ATP synthesis (p<0.01), and proton leak (p<0.05) were higher in the MOSE-L_TIC_ *_v_* than in MOSE-L spheroids. This was even more significant after reox (p<0.001 for all measurements); non-mitochondrial respiration was also significantly higher in the MOSE-L_TIC_ *_v_* spheroids (p<0.01, p<0.05, p<0.001 for 16, 20 and 24 h, respectively).

Mitochondrial respiration was very low in the spheroids confirming our previously published observations (10, 14). While the increase over time in the limiting culture conditions was statistically significant, it is unclear if these small changes are physiologically relevant. However, it is possible that only the adherent and actively outgrowing cells exhibit increased mitochondrial activity which is masked by the bulk of the spheroid containing cells with fragmented mitochondria with very low OCR and, therefore, did not drastically increase the Seahorse measurements. To determine mitochondrial adaptations in these outgrowing cells, we treated the cells with mitotracker Red before aggregation. Mitochondrial organization in the outgrowth after 24 h of adhesion of single spheroids was indistinguishable from cells grown in monolayer rather than exhibiting the fragmented phenotype we have observed in spheroids (13). Both cell types showed the round mitochondria located around the nucleus without the connected mitochondrial network; the fluorescence levels were higher in reox cells indicative of a higher mitochondrial membrane potential (**Figure 4**). This indicates that the mitochondrial fragmentation is reversible after adhesion.

We next assessed if these changes in mitochondrial organization and membrane potential in the outgrowing cells were reflected in mitobiogenic or mitophagic signaling. As shown in **Figures 4C**, the expression of PGC1α (regulator of mitobiogenesis) in outgrowing MOSE-L cells was reduced over time but were significantly elevated in reox conditions. PGC1α protein levels were significantly higher in MOSE-L_TIC_ *_v_* outgrowing cells than in MOSE-L cells at all timepoints and culture conditions (p<0.001). There was only a small reduction of PGC1α expression over time but a significantly higher expression in reox conditions (p<0.001 for all time points). In contrast, LC3 protein expression levels (regulator of mitophagy) were maintained over time in both cell lines but were significantly higher in MOSE-L_TIC_ *_v_* after 24 h (p<0.001). Reox conditions reduced LC3 expression at all timepoints (p<0.001) but the expression levels were higher in MOSE-L_TIC_ *_v_* at 16 and 24 h (p<0.001). Finally, to further observe mitochondrial dynamics and quality control in the outgrowth, we transfected the cells with MitoTimer, a fluorescent protein that localizes to the mitochondrial matrix and shifts fluorescence from green in newly generated mitochondria to red due to the dehydrogenation of the protein over time. In hypoxic conditions, both MitoTimer green (indicating mitobiogenesis) and MitoTimer red were visible in the outgrowing cells but not in the spheroid itself. The relative fluorescence of MitoTimer red or green was not changed during 16 to 24 h of adhesion in either cell line. This suggests an increased mitobiogenesis that compensates for the dilution of the transfected protein by cell division, for degradation of the protein in lysosomes *via* mitophagy or by mitochondrial proteases (38, 44) and correlates well with the increase of PGC1α in the outgrowing cells. There was no change in the ratio of MitoTimer Red/Green in MOSE-L spheroids while the ratio was reduced by the reox conditions in the MOSE-L_TIC_ *_v_* spheroids over time (20 h p<0.001, 24 h p<0.01) (**Figures 4E**). While some of the outgrowing cells exhibit only green fluorescence, suggesting these are post-mitotic daughter cells actively regenerating mitochondria, most cells show both green and red fluorescence (**Figure 4**). This is similar to reports in mouse embryonic fibroblasts that show a mixture of red and green fluorescence after 8 h in culture (45).

### Inhibition of proliferation and migration limits spheroid formation and outgrowth

**Figure 5:**
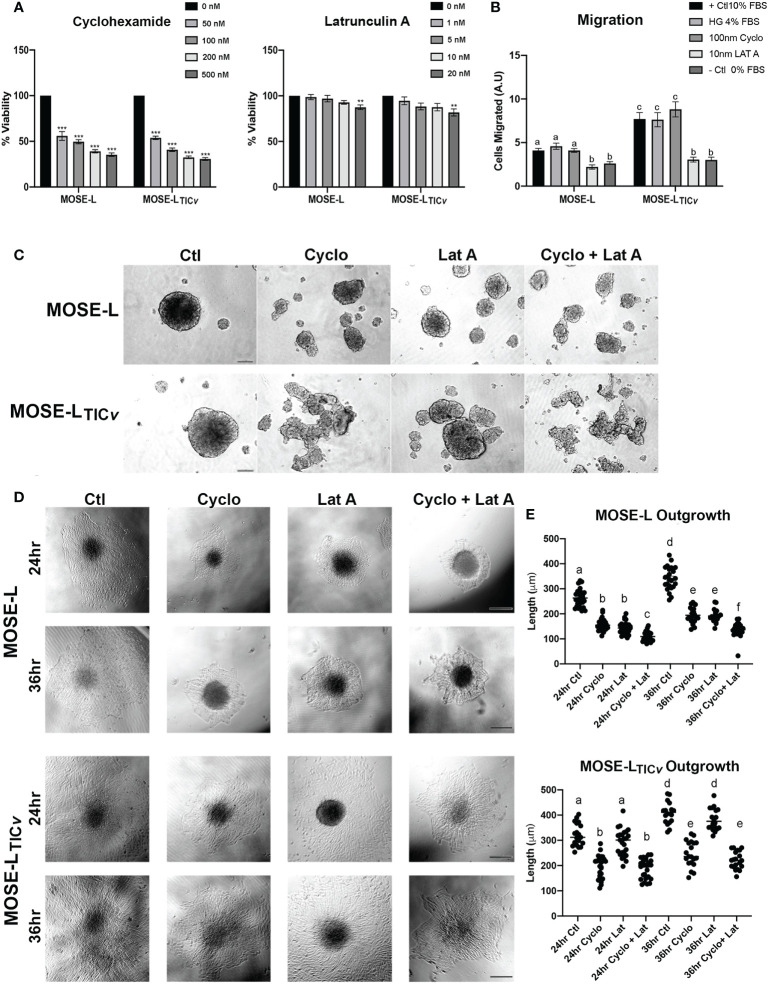
Differential effect of proliferation and migration inhibitors. **(A)** Inhibition of proliferation by treatment with Cycloheximide (50, 100, 200 and 500 nM) and Latrunculin A (1, 5, 10, and 20 nM). **(B)** Inhibition of migration after treatment with Latrunculin A (Lat A) and Cycloheximide (Cyclo) of adherent spheroids exposed to 4% FBS chemoattractant compared to positive (+; 10% FBS/4% High glucose (HG)) and negative (0% FBS) controls. Different letters indicate significant differences in cells migrating. **(C)** 24 h spheroid formation assay with 100 nM Cyclo, 10 nM Lat A or both drugs in ultra-low adherence conditions. **(D, E)** Effect of drug treatment for 24 and 36 h on spheroid outgrowth. Different letters indicate significant differences in outgrowth. Scale bar set at 200µm.

We have observed that ovarian spheroids flatten after adherence to culture plates. Considering the difference between outgrowth signaling and the primary spheroid as shown in **Figure 4**, we next determined if proliferation or migration contributed more prominently to the formation of these outgrowth structures. To avoid the influence of apoptosis on our measurements, we first determined the toxicity of the protein translation inhibitor Cycloheximide (cyclo) that inhibits proliferation and the actin stabilizer Latrunculin A (Lat A) that inhibits motility. Cyclo treatment at a concentration of 50 to 500 nM effectively reduced proliferation (doubling times of the MOSE cells are ~11 h) but did not induce cell death; 10 nM Lat A preserved more than 80% viability compared to the control (**Figure 5**). We then treated the cells with the drugs for 24 h before spheroid formation. Interestingly, both drugs individually and combined prevented the formation of a single spheroid; instead, multiple small spheroids were formed after 24 h (**Figure 5**). Since we had previously observed that smaller spheroids more rapidly flatten onto tissue culture dishes than larger ones (unpublished observations), the drugs were added to the media only after spheroid formation to eliminate the impact of spheroid size differences on the functional assays. To confirm the specificity of the inhibitors on proliferation and migration, we plated the spheroids into Boyden chambers, treated them with the drugs for 6 h and determined the effects on cell migration. As shown in **Figure 5**, there was no difference in migrating cells between the positive control (HG DMEM, 10% FBS) and the controls grown in regular growth medium (HG DMEM, 4% FCS), indicating the 4% FBS is sufficient as chemoattractant while the negative control that contained no FBS induced little migration. 10 nM Lat A significantly suppressed the migration of both MOSE-L and MOSE-L_TIC_ *_v_* spheroids compared to the controls (p<0.001) while the treatment with 100 nM cyclo had no effect. The combination of drugs did not reduce the number of migrating cells beyond the effect of Lat A. We then plated the spheroids onto tissue culture dishes to assess the impact of the drug treatment on the outgrowth capacity. Both drugs suppressed outgrowth in the MOSE-L after 24 and 36 h (p<0.05) without a significant difference between the treatments. The suppression of outgrowth was significant higher with the combination treatments (p<0.001 for both treatments and time points). In contrast, only cyclo treatment suppressed MOSE-L_TIC_ *_v_* outgrowth compared to the controls (p<0.001 for both time points) while Lat A treatment had no effect (p=0.411, p=0.726 for 24 and 36 h, respectively) (**Figures 5D**). Since the combined treatment of cyclo plus Lat A was not significantly different from the cyclo treatment alone but significantly lower than the Lat A treatment alone (p<0.001 at both time points), the data indicate that the reduced outgrowth capacity is due to the effect of the cyclo in MOSE-L_TIC_ *_v_*. Together, these results indicate the spheroidal outgrowth capacity in MOSE-L spheroids is reliant on both motility and proliferation but is driven by proliferation in the MOSE-L_TIC_ *_v_* spheroids.

### Inhibition of proliferation and migration changes mitochondrial quality control and metabolic functions

**Figure 6:**
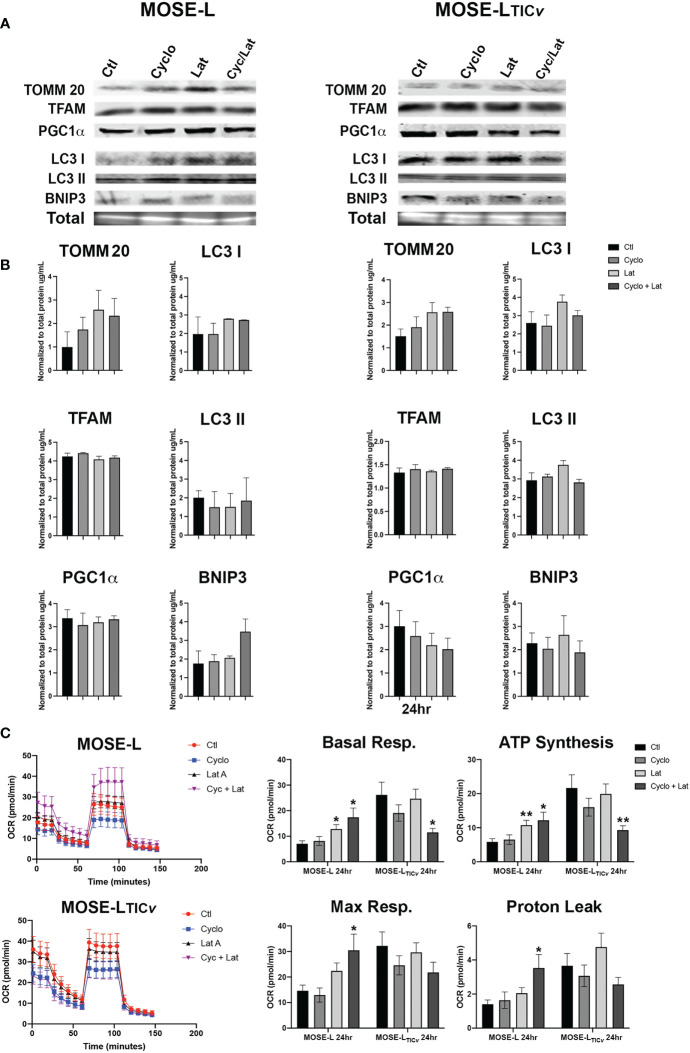
Effect of drug treatment on mitochondrial quality control and respiration. **(A, B)** Western blot analyses of mitobiogenic (TOMM20, TFAM and PGC1α) and mitophagic (LC3 I, II, and BNIP3) protein expression after 24 h treatment with100 nM Cycloheximide (cyclo), 10 nM Latrunculin A (Lat A), or both. **(C)** Seahorse XFe96 analysis of cellular respiration (OCR, oxygen consumption rate); *p<0.05, **p<0.01 *vs*. control.

Next, we assessed if the drug treatments modulated the expression of mitochondrial regulators. We observed a slight increase in mitochondrial content protein TOMM20 at 24 h of incubation with Lat A in both cell lines (**Figures 6A**). Treatment of the spheroids with cyclo, Lat A or a combination had little effect on the expression of proteins regulating mitobiogenesis or mitophagy. We saw a decrease in PGC1α in the MOSE-L_TIC_ *_v_* spheroids albeit this was not significant (**Figures 6A**). The ratio comparing mitophagy:mitobiogenesis protein expression were not changed suggesting that neither drug treatment caused a shift towards mitobiogenesis or mitophagy. Metabolic adaptations to the drugs showed differential responses in both cell types. The MOSE-L spheroids show an increased basal and maximal respiration and ATP synthesis rate after treatment with Lat A treatment compared to the untreated controls (p<0.05) that was more pronounced in the combination group (p<0.01). This was accompanied by an increased proton leak (p<0.05) in the combined treatment group (**Figure 6**). There was no effect of cyclo on cellular respiration in MOSE-L. In contrast, Lat A and the combinatorial treatment with cyclo showed a reduction in basal respiration (p<0.05), ATP synthesis (p<0.01), and proton leak in the MOSE-L_TIC_ *_v_* spheroids while Lat A and cyclo treatment alone had little effect on cellular respiration. These data indicate that treatment with the drugs differentially modulated mitochondrial respiration; however, as mentioned above, the OCR and drug-indued changes were very low and likely masked by the bulk of the spheroid and their physiologic relevance remains unclear.

### Spheroidal outgrowth is primarily produced *via* proliferative signaling from different regions of the primary spheroid

**Figure 7:**
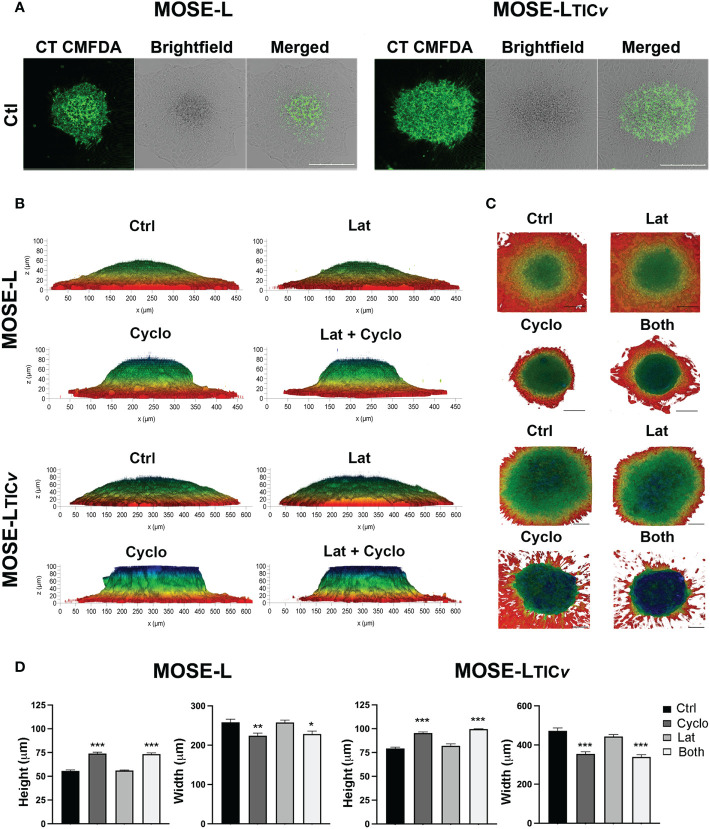
Spheroidal outgrowth results from proliferative and migratory signaling. **(A)** Confocal images of spheroids outgrowth layer from cell-tracker CMFDA stained spheroids adhered for 24 h were treated with Cyclo and Lat A **(A)** Spheroids outgrowth layer from cell-tracker CMFDA stained spheroids adhered for 24 h treated with Cyclo and Lat A **(B)** Side-view profiles of drug-treated spheroids observed through 3D reconstruction of 100 µm optical z-stacks using the Leica LASX software depth-coded to show z-profiles. **(C)** Top-view perspectives of spheroidal spread and outgrowth production depth coded to show z-profiles. Scale bar set at 200µm. **(D)** Quantification of height and width (MOSE-L and MOSE-LTIC*v*) of spheroids from **(B)** using ImageJ. *p<0.05, **p<0.01, ***p<0.001 treatment compared to the control group.

To further investigate the importance of proliferation vs. migration for spheroidal outgrowth, we next stained the spheroids with cell tracker CMFDA prior to adherence for 24 h. Since the CMDFDA dye dilutes in response to mitotic events (46), we assessed if the spheroidal outgrowth was reflective of proliferation based on the fluorescence intensity of the outgrowth compared to the spheroid using confocal microscopy. Regardless of the drugs used, the cells in the outgrowth retained very little fluorescent signals compared to the adherent core spheroid cells in both MOSE-L and MOSE-L_TIC_ *_v_* (**Figure 7**) indicating the dilution of the dye due to cell division. This confirms the observations above that proliferation is important for the outgrowth of both cell lines (see **Figure 4**). We next determined if treatment with either drug or their combination would affect the adhesion and the flattening of the spheroids after adhesion. The 3D reconstruction software from the Leica DMI8 MP confocal microscope and depth coding analysis allowed for determining the shape of the adhering spheroids. MOSE-L spheroids were generally smaller and therefore the diameter of the spheroid is less than MOSE-L_TIC_ *_v_* spheroids (**Figures 7B**); nonetheless, as shown in **Figure 1**, the outgrowth diameter was similar to that of the MOSE-L_TIC_ *_v_* spheroids. Treatment with Lat A did not affect the flattening of the spheroids of either cell line as neither the height nor the width of the spheroids was different from the untreated controls (**Figures 7B**). In contrast, treatment with cyclo effectively prevented the flattening of the spheroids with no difference to the combination treatment (**Figures 7B** suggesting a need for the synthesis of proteins to allow for the contour change.

### Inhibition of protein translation with cycloheximide suppresses invasion *via* inhibition of collagenase and MMP protein expression

**Figure 8:**
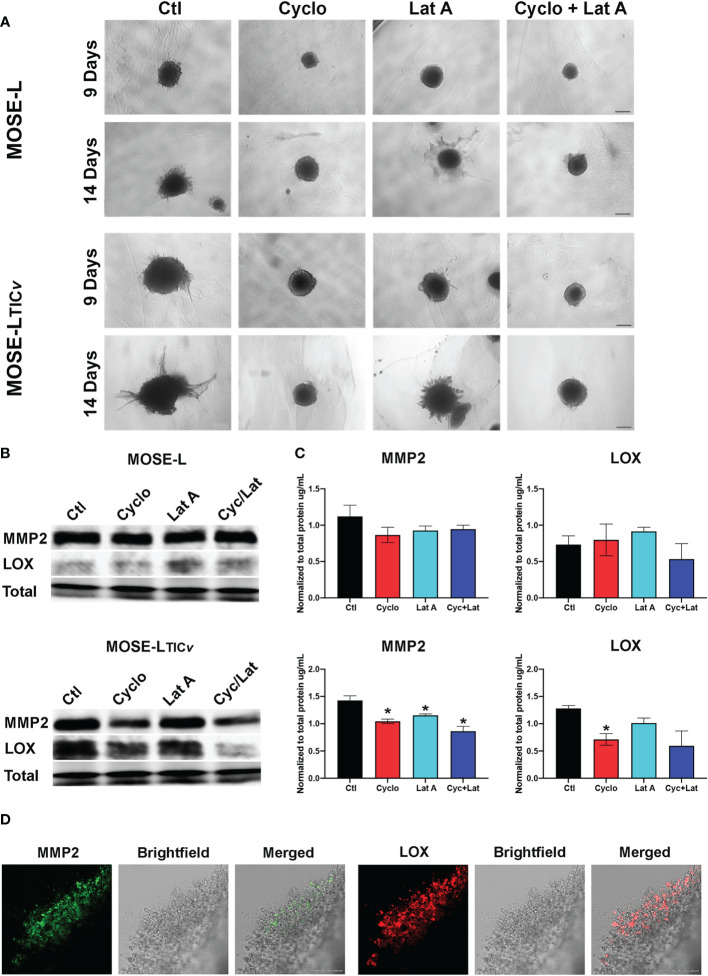
Inhibition of protein synthesis limits invasion through decreased MMP and LOX expression. **(A)** Spheroids embedded in collagen gels were treated with Cycloheximide (cyclo), Latrunculin A (Lat A), or a combination for 9 and 14 days. **(B, C)** Western blot analysis of MMP2 and LOX proteins in 24 h drug-treated adherent spheroids; *p<0.05 compared to the untreated control. **(D)** Embedded MOSE-L_TIC_ *_v_* spheroids after 9 days were immunostained for MMP2 and LOX antibodies. Scale bar set at 200µm.

To assess how the drug treatment affects the invasive capacity, we embedded the MOSE-L and MOSE-L_TIC_ *_v_* spheroids in collagen and then added cyclo, Lat A, or a combination treatment with both drugs to the medium for 9 or 14 days. As shown in **Figure 8**, treatment with cyclo or the combined treatment suppressed spheroid growth and prevented the invasion of the collagen. Lat A, however, had less of an inhibitory effect on the growth of the embedded spheroids or the invasive capacity in both spheroid models. To determine if this observed limited invasive capacity resulted from decreased extracellular matrix remodeling activity, as a proof-of-concept we next determined MMP2 and LOX protein expression in drug-treated spheroids (**Figures 8B**) since these proteins are associated with ovarian cancers’ invasive capacity (47–49). We found a similar expression of both MMP2 and LOX in the MOSE-L spheroids. Treatment with either drug caused a significant decrease in MMP2 protein expression in the MOSE-L_TIC_ *_v_* spheroids (p<0.05) but had little effect on MMP2 expression in the MOSE-L spheroids. Additionally, only cycloheximide-treated MOSE-L_TIC_ *_v_* spheroids showed a significant decrease in LOX expression (p<0.05) (**Figure 8**); the lower expression after combination treatments were not statistically significant. Lastly, both MMP2 and LOX proteins were expressed in the invasive areas rather than in the compact spheroids (**Figure 8**). Thus, the bulk of the unresponsive spheroid cells may mask the impact on the protein expression levels These data indicate that the reduced invasive capacity is associated with a lower expression of ECM remodeling enzymes and suggest that inhibition of proliferation with cyclo also reduced the expression of proteins important for invasion.

## Discussion

Ovarian cancer cells aggregate during or after exfoliation which enhances their viability during dissemination in the highly hypoxic and nutrient-starved peritoneal cavity. We have previously shown that mitochondria in ovarian cancer spheroids represent a more fragmented morphological phenotype that is highly mitophagic (13), exhibits reduced oxidative phosphorylation and, consequently, a severely reduced energy production (10). However, to support adhesion and invasion of their metastatic sites, the cancer cells need to produce more energy. Thus, we hypothesized that the fragmented mitochondrial phenotype of the ovarian cancer cells is reversible in contrast to cardiomyocytes and other benign tissues where fragmentation induced cell death (50). For the present proof-of-concept studies we used our syngeneic MOSE model for progressive serous ovarian cancer that has been genetically and functionally correlated to the human disease (34, 35). This model prevents that inter-individual differences that occur with differing cell lines affect our comparisons between slow- and fast-developing disease and characterize an aggressive phenotype. Here we determined differences in the mitochondrial plasticity of slow and fast-developing ovarian spheroids focusing on early adhesion events, and how an adaptation to a changing environment contributes to invasive and proliferative functions. We then simulated nutrient and oxygen restoration following early adherence (access to existing blood vessels or angiogenesis) by changing to culture conditions with high nutrient and oxygen levels. We show that upon adhesion, all cells adhere to and grow onto the culture dishes even in limiting conditions. Adhesion was associated with the restoration of mitochondrial morphology in the outgrowing cells, indicating that the mitochondrial fragmentation was indeed reversible and activated by adhesion signals and, thus, limited to the cells actually touching the culture dish and not observed in the bulk of the spheroid. Although still very low, adhesion increased the oxidative capacity of both cell lines but much more so in the aggressive MOSE-L_TIC_ *_v_*. It possible that this upregulation of respiration and ATP production was limited to the adherent cells with the restored phenotype and our measurements were masked in by the bulk of the unresponsive spheroid cells. This is supported by our observations that the outgrowing cells express significant levels of PDC1α, a master regulator of cellular mitobiogenesis. Adhesion caused an increase in mitochondrial mass but little change in the mitochondrial dynamics after 16 h of adhesion as the ratio of mitobiogenesis and mitophagy regulators did not change at that timepoint.

Only the less aggressive MOSE-L cells responded to reox conditions with enhanced proliferation and migration and an increase mitobiogenic and a decrease in mitophagic protein expression. In contrast, proliferation and outgrowth was unaffected by the oxygen and nutrient levels in the aggressive MOSE-L_TIC_ *_v_*; while both spheroid types showed an increase in oxidative phosphorylation after reox, this was significantly higher in MOSE-L_TIC_ *_v_* spheroids and reox caused a shift of the mitochondrial regulatory proteins towards mitophagy rather than to mitobiogenesis that was observed in the MOSE-L spheroids. These changes did not affect the proliferation of MOSE-L_TIC_ *_v_* spheroids but increased their invasive capacity while only the MOSE-L also increased their migration after reox. Then we investigated the underlying processes driving the outgrowth and found that both proliferation and migration contributed to the outgrowth of MOSE-L spheroids but mostly proliferation drove the outgrowth of the MOSE-L_TIC_ *_v_*. Consequently, the inhibition of protein synthesis reduced proliferation, flattening of the spheroids and the invasion of a collagen gel. This suggests that both the flattening of the spheroids and the following invasion are active processes that could be supported by the upregulation of ATP production in adherent cells or the collagen-associated peripheral cells to synthesize the necessary proteins. This was confirmed by the expression of MMP2 and LOX in collagen-embedded spheroids and may be driven by adhesion signals activated by the collagen ligation of surface proteins such as integrins. The morphological and functional changes in the spheroid cells may be increased after prolonged adhesion when due to the flattening of the spheroids the adhesion signals may affect more cells; the underlying adhesion signals and their timeline will be determined in our future studies.

Mitobiogenesis increases mitochondrial mass and has been linked to cancer cell proliferation and survival (29–31) as well as to tumorigenic support of metabolic adaptations and growth (29, 30, 51). Its key regulator PGC1α was upregulated in highly oxidative tumors (52, 53) and promoted invasion and migration but did not affect proliferation (32). We show here that PGC1α was expressed in the outgrowing cells; nuclear localization was enhanced by reox conditions which correlates well with the increase in respiration and outgrowth. Yet, hypoxia-mediated mitophagy is often upregulated in tumors including lung, breast, and other cancers (21, 54), inducing a metabolic switch to glycolysis to support viability when nutrients are scarce (55). Human ovarian tumors show a high overrepresentation of mitophagy pathways (56). A recent report concluded that mitophagy is required for the adaptive reprogramming of cellular metabolism (57), and provides molecules for cell survival and enhanced stemness and stem cell renewal (see review (58). Further, BNIP3 has been shown to support migration *via* maintaining actin cytoskeleton plasticity and focal adhesion dynamics (59), linking hypoxia-induced mitophagy with cellular functions. Interestingly, in contrast to the MOSE-L which showed a decrease in the mitophagic and autophagic proteins BNIP3/LC3 I and II, we saw a 2-fold increase in these proteins in the MOSE-L_TIC_ *_v_* spheroids during reox. This correlates well with the apparent importance of mitophagy in these aggressive cells. Further, the relative stability of MitoTimer expression suggests that mitophagy is counterbalanced by mitobiogenesis and suggests a better mitochondrial quality control in the MOSE-L_TIC_ *_v_*. These results indicate that adhesion signals differentially direct mitochondrial biogenesis and quality control in cells representing slow- or fast-developing disease that an increase in mitophagy that increases mitochondria quality may contribute to the aggressive phenotype. The signaling cascades involved in these cells and their potential as therapeutic targets are currently investigated in our laboratory.

Spheroid formation has been attributed to an increase in cytoskeletal rearrangements, specifically within actomyosin contractile forces (47). Interestingly, we find that spheroid formation is impeded by both inhibition of proliferation and migration, suggesting that aggregation is not a passive process but requires protein synthesis and motility. Cancer spheroids have a layered cellular organization where only the outer shell contains proliferative cells while the inner layers are more quiescent (10). We have shown that only the outer layer of the ovarian spheroids contains visible mitochondria and that a reduced proliferation and respiration may key factors to the survival of the spheroids (13). Disseminating ovarian spheroids attach to the mesothelium covering the peritoneal organs aided by fibronectin released from the mesothelial cells (60) or the cancer cells themselves (35). The flattening of the spheroids onto their substrate is also an active process that requires the synthesis of proteins since cyclo significantly inhibited flattening. Ovarian cells then use acto-myosin-generated forces to breach the mesothelial layers (61) and secrete enzymes to remodel and invade the extracellular matrix underneath. Adhesion signals induced MMP2 expression in the outgrowing cells and the peripheral spheroid cells in contact with collagen, confirming reports that show that MMP2 expression is an early event in secondary metastasis (48) and that the inhibition of MMP2 can reduce the number of metastases *in vivo* (49). Further, LOX expression has been corelated to the invasive capacity of ovarian metastases (47). Similarly, suppression of protein synthesis reduced MMP2 and LOX expression and reduced invasion in our model. Our data indicate that slow-developing ovarian cancer cells require access to oxygen to support initial invasion while the aggressive MOSE-L_TIC_ *_v_* are less affected by hypoxia.

The driving force behind outgrowth signaling has been suggested to be a product of actin cytoskeletal rearrangement (62) as well as decreased E-cadherin expression (63). The cause of outgrowth signaling in adherent ovarian cancer spheroids has not yet been elucidated, however, it has been suggested that stabilized adhesion is controlled in part by ECM deposition (64, 65), integrin signaling (66, 67), and oncogene-induced fast growth (9). Here we show for the first time the differential outgrowth signaling between less and highly aggressive cells. The MOSE-L cells relied on both proliferation and motility to generate outgrowth while our highly aggressive MOSE-L_TIC_ *_v_* were more reliant on proliferation. This suggests that the cells from the outer layers of the spheroids are actively proliferating upon adhesion; the flattening of the spheroids can generate a larger adhesion layer that allows for the re-organization of mitochondria. This agrees well with our previous observations that glucose uptake was only drastically increased in the adherent spheroid cells and their outgrowth (14) and indicates that the fragmented mitochondrial phenotype is indeed reversible after adhesion.

In summary, this study shows the reversal of mitochondrial fragmentation by adhesion signals and simulated reoxygenation. We demonstrated that changes in mitochondrial dynamics support proliferative, migratory and invasive functions of the cancer cells, and metabolic adaptations specifically within the spheroidal outgrowth. We found that the aggressive cancer phenotype is characterized by a higher reliance on mitophagy but lower reliance on oxygen and nutrients and preservation of proliferative, invasive, and migratory functions even in non-permissive growth conditions compared to our slow-developing MOSE-L spheroid model. Further, the aggressive cells rely mostly on proliferation for outgrowth after adhesion. These results provide the proof-of-concept that adhesion directs the reversal of the fragmented mitochondrial morphology and up-regulates ATP synthesis necessary for post-adhesion events such as migration and invasion. Understanding how these key processes are regulated could provide new preventative targets directly associated with mitochondrial plasticity, quality control and metastatic processes to suppress the metastatic capacity of highly aggressive ovarian cancer spheroids. These adhesion signals are currently the focus of our investigations.

## Data availability statement

The raw data supporting the conclusions of this article will be made available by the authors, without undue reservation.

## Author contributions

JG and ES wrote the manuscript. JG and ES contributed to design and conception of experiments. JG, ES, SC, NB, LB, and AN were all involved in data collection, analysis, and interpretation of collected datasets. ES and JD provided technical and supporting materials for experimentation. JG and ES contributed to interpretation and in-depth analysis of data. ES supervised the study. All authors were involved in reviewing and editing manuscript and figures prior to submission of this article.

## Supplementary material

Supplementary Figure 1 Changes in spare respiratory capacity, glycolytic reserve and non-mitochondrial respiration after reoxygenation. p<0.05 to hypoxic controls.
