# Supplementary figures and images for: Mitochondrial plasticity supports proliferative outgrowth and invasion of ovarian cancer spheroids during adhesion
Source: Front Oncol. 2023 Jan 16;12:1043670. doi: 10.3389/fonc.2022.1043670 (PMC9884807; doi:10.3389/fonc.2022.1043670)

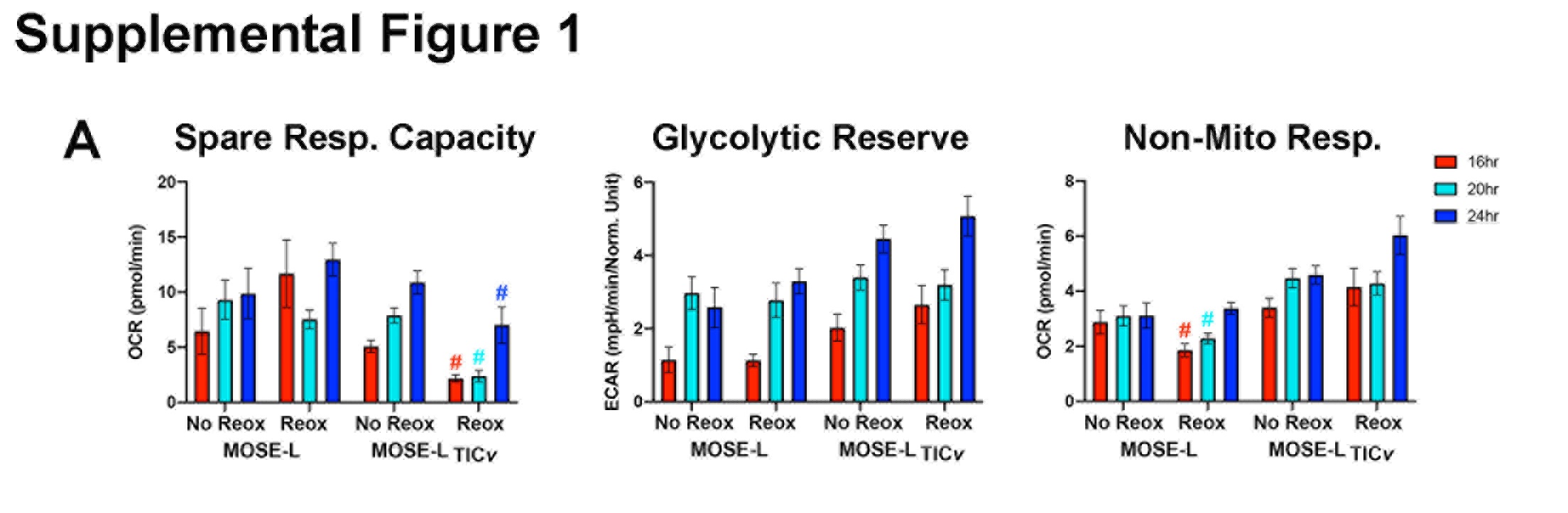

Supplement: Supplementary Figure 1 — Changes in spare respiratory capacity, glycolytic reserve and non-mitochondrial respiration after reoxygenation. p<0.05 to hypoxic controls. [file Image_1.jpg]
